# Daptomycin for the Treatment of Gram-Positive Periprosthetic Hip Infections: Can Daptomycin Prevent the Implant Removal?

**DOI:** 10.7759/cureus.15842

**Published:** 2021-06-22

**Authors:** Kenichi Oe, Masahiro Sawada, Tomohisa Nakamura, Hirokazu Iida, Takanori Saito

**Affiliations:** 1 Department of Orthopaedic Surgery, Kansai Medical University, Hirakata, JPN

**Keywords:** daptomycin, periprosthetic joint infection, implant retention, infection control rates, preoperative risk scores

## Abstract

Introduction

Management of periprosthetic hip infections (PHIs) generally consists of implant removal and thorough debridement, accompanied by appropriate antibiotic therapy. Daptomycin (DAP) is a novel antibiotic, which allowed for implant retention in several patients after treating their infected joints. However, there is no consensus about implant retention or removal during the treatment of PHIs. The aim of this study was to examine the effect of DAP and to determine a surgical treatment strategy.

Methods

This study retrospectively evaluated 20 patients between August 2014 and December 2018, divided into implant retention (n=9) and implant removal groups (n=11). Infection control and risk of recurrent infection were evaluated. Infection control was defined as not requiring implant removal after the final treatment.

Results

Infection control rates in implant retention and implant removal groups were 67% and 90%, respectively. All late chronic infections resulted in failure cases within the implant retention group. In the implant retention group, mean preoperative risk scores for successful cases were significantly higher than those for failure cases (p<0.05).

Conclusions

Patients with low risk did not require implant removal, suggesting that DAP may be a breakthrough alternative to traditional PHI management.

## Introduction

The gold standard for managing periprosthetic joint infection (PJI) generally consists of implant removal and thorough debridement, accompanied by appropriate antibiotic therapy. According to the guidelines for the management of PJIs by the Infectious Diseases Society of America (IDSA) [1], the probability of implant retention is limited; only patients diagnosed with a PJI who (1) have a well-fixed prosthesis without a sinus tract and (2) are within approximately 30 days of prosthesis implantation or less than three weeks from the onset of infectious symptoms should be considered for debridement and retention of the prosthesis. However, there is no consensus for the treatment of PJIs regarding the choice of specific antibiotic therapy.

Daptomycin (DAP) could an option for PJI treatment because it exerts bactericidal activity against Gram-positive bacteria, including multiple-resistant isolates, and stationary-phase bacteria in biofilm present on implants [2]. DAP is currently available as a novel antibiotic therapy worldwide; the European Registry demonstrated high clinical success in PJI treatment with DAP therapy, including implant retention in 56% of patients [3]. However, PJI treatment with implant retention remains controversial and challenging. The aim of this study was to examine the effect of DAP on implant retention in patients with periprosthetic hip infection (PHI) and to determine a surgical treatment strategy. The hypothesis of the study was that DAP would prevent implant removal for patients with low-risk PHIs.

This article was previously published in a preprint server (https://www.researchsquare.com/article/rs-6833/v1).

## Materials and methods

Study population

**Figure 1:**
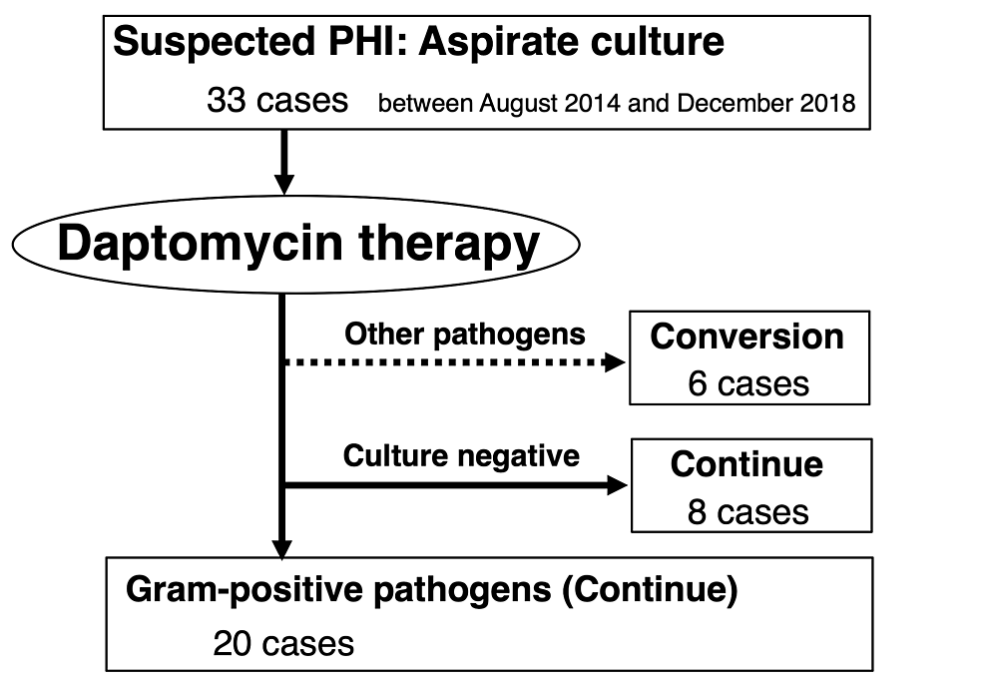
Study flowchart. PHI, periprosthetic hip infection.

Between August 2014 and December 2018, 33 patients provided with DAP (Cubicin®, MSD K.K., Tokyo, Japan) as a treatment for a PHI at our institution. DAP was administered when an infection was suspected, right after aspiration of the joint for subsequent cell culture. However, some of those patients changed the antibiotic treatment to a more appropriate one after the identification of the pathogen. In cases where no identification was made by joint aspiration, DAP therapy was initiated, and if the C-reactive protein (CRP) levels decreased, the treatment continued. Thus, a retrospective study for the treatment of infections caused by Gram-positive pathogens with DAP was conducted in only 20 patients (follow-up rate of 100%; Figure 1). In accordance with the IDSA guideline [1], the implant retention was initially selected, but the final decision was made intraoperatively by the surgeons. Rifampicin (RFP) was added whenever possible, and other antibiotics were not used with DAP therapy.

Surgical options included implant retention in 9 patients (no surgery in four and only debridement in five) and implant removal in 11 patients (one-staged and two-staged revisions in three and eight, respectively). The implant retention group included three men and six women, with a mean age of 69 years (range: 36-85 years) and a mean follow-up period of 24 months (6-39 months). The mean duration of DAP therapy was 30 days (12-106 days), at a mean daily dose of 5.6 mg/kg/day (3.8-8.3 mg/kg/day). The implant removal group included four men and seven women, with a mean age of 69 years (53-88 years) and a mean follow-up period of 23 months (4-50 months). The mean duration of DAP therapy was 37 days (1-60 days), at a mean daily dose of 5.8 mg/kg/day (3.8-10.0 mg/kg/day; Table 1). During DAP therapy, adverse events including increased creatine phosphokinase or decreased renal function were monitored. After DAP therapy, we switched to oral antibiotics depending on the pathogen and susceptibility when the decreased CRP level was continued. The study was approved by our institutional review board. All patients provided informed consent for study participation and publication of findings.

**Table 1:** Preoperative patient characteristics. ^a^Student t-test. ^b^Fisher exact test. ^c^Mann-Whitney U test.

Characteristics	Implant retention	Implant removal	p-value
Number of joints	9	11	
Mean age at surgery, years (range)	69 (36–85)	69 (53–88)	0.580^a^
Gender, male/female	3:6	4:7	0.630^b^
Mean follow-up period, months (range)	24 (6–39)	23 (4–50)	0.061^a^
Number of patients previously administered with other antibiotics	7	9	0.625^b^
Mean dose, mg/kg/day (range)	5.8 (3.8–8.3)	5.8 (3.8–10.0)	0.313^a^
Mean duration of daptomycin therapy, day (range)	30 (12–106)	37 (1–60)	0.095^c^
Number of patients administered with rifampicin	7	8	0.604^b^
Infected implant			0.579^b^
Bipolar hip arthroplasty	1	2	
Total hip arthroplasty	8	9	
Surgical intervention during this therapy, number			
None	4	0	0.026^b^
Only debridement	5	0	0.008^b^
One-staged revision	0	3	0.145^b^
Two-staged revision	0	8	0.001^b^

Bacterial infection diagnosis

PHI was diagnosed according to the criteria of the Musculoskeletal Infection Society [4]. PHI was classified into four clinical categories: type I (early postoperative infection), type II (late chronic infection), type III (acute hematogenous infection), and type IV (positive intraoperative cultures) [5,6]. An early postoperative infection was defined as a wound infection that developed less than one month after surgery. A late chronic infection corresponded to an infection that developed one month or more after the index operation and that had an insidious clinical course. Acute hematogenous infection was associated with a documented or suspected antecedent bacteremia and was characterized by an acute onset of symptoms in the affected joint with the prosthesis. A patient was considered to be in the type IV group if at least two specimens obtained at the time of revision surgery were positive on culture.

Patients follow-up

After antibiotic administration, patients were followed up at weeks: 1, 2, 3, 4, 8, and 12, at months 6 and 9, at one year, and annually thereafter. Data were retrospectively analyzed by two orthopedic surgeons who were blinded to the treatment regimens. Pathogens causing PHIs, reasons for the discontinuation of an antibiotic, and infection control rates were evaluated. Infection control was defined as the lack of clinical signs, symptoms, and radiological signs of infection, a CRP level <10 mg/L, an erythrocyte sedimentation rate <20 mm/h, and not requiring implant removal after the final treatment. Therefore, a successful case was defined as one not requiring implant removal after treatment; failure was defined as implant removal due to recurrent infection.

Clinical parameters

**Figure 2:**
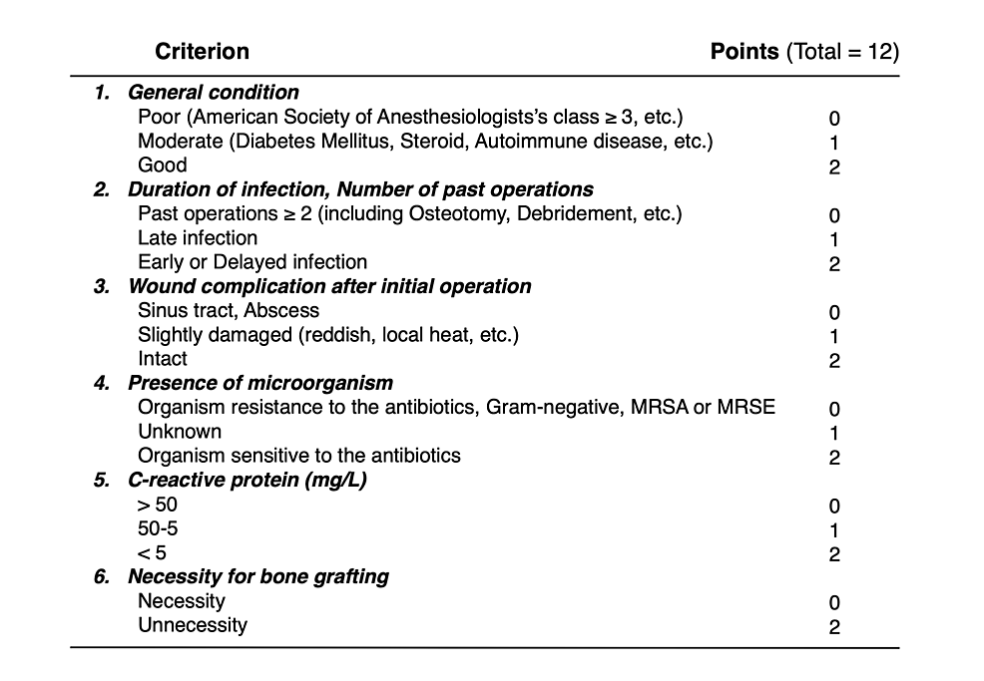
Pre-operative scoring system to assess the risk of recurrent infection. Each parameter was rated from 0 to 2 points, giving a maximum score of 12 points for low-risk.

For the laboratory assessment, CRP levels (mg/L) were investigated. Furthermore, the risk of recurrent infection was evaluated using the scoring system (Figure 2) [7], based on six parameters: (1) general condition, (2) duration of infection, (3) wound complication, (4) presence of microorganisms, (5) CRP levels, and (6) necessity for bone grafting. Each parameter was rated from 0 to 2 points, giving a maximum score of 12 points for low-risk.

Statistical analysis

Two-group comparisons were conducted using the Student t-test or Mann-Whitney U test. To compare qualitative variables, Fisher's exact test was applied. The Wilcoxon signed-rank test was used to compare differences in parameters before and after patient treatment. Statistical significance was defined as p<0.05. All analyses were performed using SAS 9.2 (SAS Institute Inc., Cary, NC, USA).

## Results

The summary of results for each patient is shown in Table 2. In one patient (case 14), DAP was discontinued because of an anaphylactic shock, and statistical analyses were performed except in case 14. Other adverse events have not occurred. Infection control rates in the implant retention and implant removal groups were 67% (6/9) and 90% (9/10), respectively. Clinical success rates of the implant removal group were significantly higher than those of the implant retention group (p<0.05). In case of no methicillin-resistant bacteria, infection control rates in the implant retention and implant removal groups were 60% (3/5) and 67% (2/3), respectively. In the case of methicillin-resistant bacteria, infection control rates in the implant retention and implant removal groups were 75% (3/4) and 100% (7/7), respectively. In the implant retention group, successful cases experienced type I (three patients) and type III (three patients) infections, whereas failure cases only presented type II infections (three patients). The mean daily dose of DAP (mg/kg/day) in the implant retention group was 5.0 (3.8-5.9) and 7.1 (5.9-8.3) for successful and failure cases, respectively. Similarly, in the implant removal group, DAP daily dose was 5.9 (3.8-10.0) and 5.8 for successful and failure cases, respectively.

**Table 2:** Characteristics and results of each patient infected with Gram-positive pathogens before and after DAP treatment. DAP: daptomycin, THA: total hip arthroplasty, BHA: bipolar hip arthroplasty, CNS: coagulase-negative *Staphylococcus*, MRCNS: methicillin-resistant coagulase-negative *Staphylococcus*, MRSE: methicillin-resistant *Staphylococcus epidermidis*, MRSA: methicillin-resistant *S. aureus*, MSSE: methicillin-sensitive *S. epidermidis*, MSSA: methicillin-sensitive *S. aureus*. ^a^Infection type: type I (early postoperative infection), type II (late chronic infection), type III (acute hematogenous infection), and type IV (positive intraoperative cultures).

Case	Age/gender	Infected implant	Pathogen	Infection type^a^	Duration of DAP (day)	DAP daily dose (mg/kg/day)	Preoperative scoring	Surgical intervention	Additional surgery or adverse event
1	81/female	THA	Streptococcus sp.	III	15	4.2	9	No surgery	(-)
2	75/female	THA	CNS	III	16	5.1	8	No surgery	(-)
3	77/female	BHA	MRCNS	I	12	5.3	7	No surgery	(-)
4	62/female	THA	MRSE	II	14	8.3	7	No surgery	Two-staged revision
5	36/male	THA	MRSE	I	27	5.9	8	Only debridement	(-)
6	64/male	THA	Staphylococcus sp.	III	36	5.7	8	Only debridement	(-)
7	85/male	THA	MRSA	I	27	3.8	7	Only debridement	(-)
8	64/female	THA	Streptococcus sp.	II	19	5.9	6	Only debridement	Two-staged revision
9	75/female	THA	CNS	II	106	7.0	6	Only debridement	Two-staged revision
10	76/female	THA	Streptococcus sp.	II	49	7.6	10	One-staged revision	(-)
11	88/male	BHA	MRSE	III	22	5.3	8	One-staged revision	(-)
12	82/female	THA	MSSE, CNS	II	31	3.8	7	One-staged revision	(-)
13	62/female	THA	MSSE	I	42	6.0	8	Two-staged revision	(-)
14	55/female	THA	MSSA	I	1	4.4	8	Two-staged revision	Anaphylaxis
15	66/male	BHA	MRSE	I	60	4.5	7	Two-staged revision	(-)
16	53/male	THA	MSSA	II	25	4.9	7	Two-staged revision	(-)
17	62/male	THA	CNS	II	31	5.0	7	Two-staged revision	(-)
18	65/female	THA	MRSE	II	57	10.0	6	Two-staged revision	(-)
19	74/female	THA	MRSA	II	48	5.8	6	Two-staged revision	(-)
20	74/female	THA	Streptococcus sp.	II	44	5.8	5	Two-staged revision	Two-staged revision

**Figure 3:**
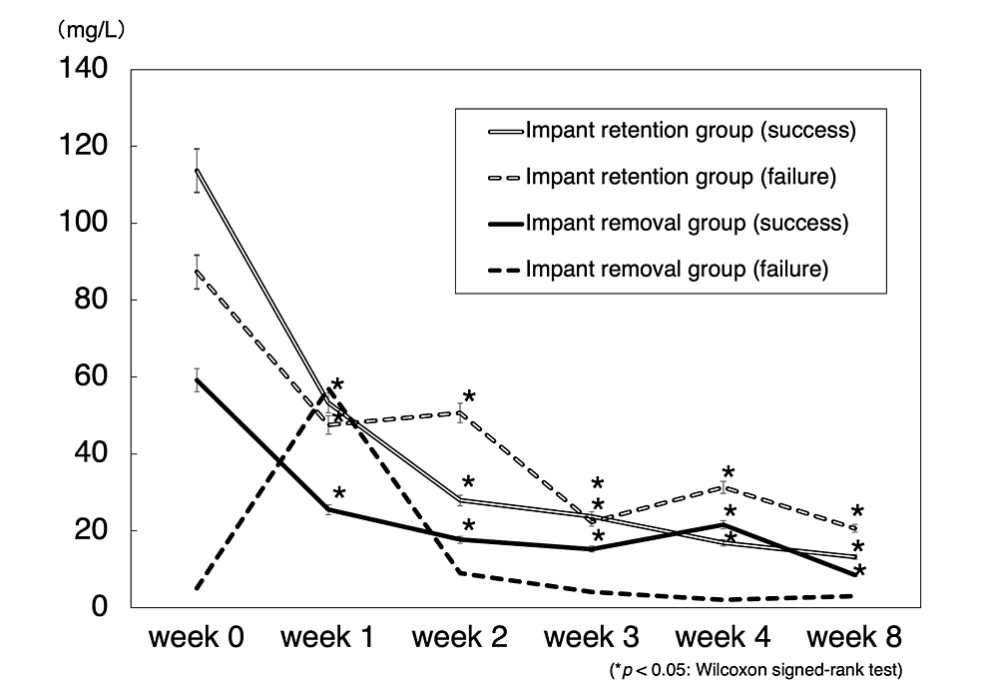
Mean C-reactive protein concentration in patients infected with Gram-positive bacteria and treated with DAP. The Wilcoxson signed-rank test was used to compare differences before and after treatment of patients. Data are expressed as the means and two-sided 95% confidence interval.

The mean CRP values for each group were recorded for eight weeks, and the results were grouped according to the outcome of the DAP treatment (success or failure) for each group (Figure 3). CRP levels in all cases, except for the failure case in the implant removal group, were significantly decreased. Mean preoperative risk scores of the implant retention group were 7.8 points (7-9 points) and 6.3 points (6-7 points) for successful and failure cases, respectively. Mean preoperative risk scores for successful cases were significantly higher than those for failure cases (p<0.05). In the implant removal group, the score for success and failure cases were 7.3 points (6-10 points) and 5.0 points, respectively.

**Figure 4:**
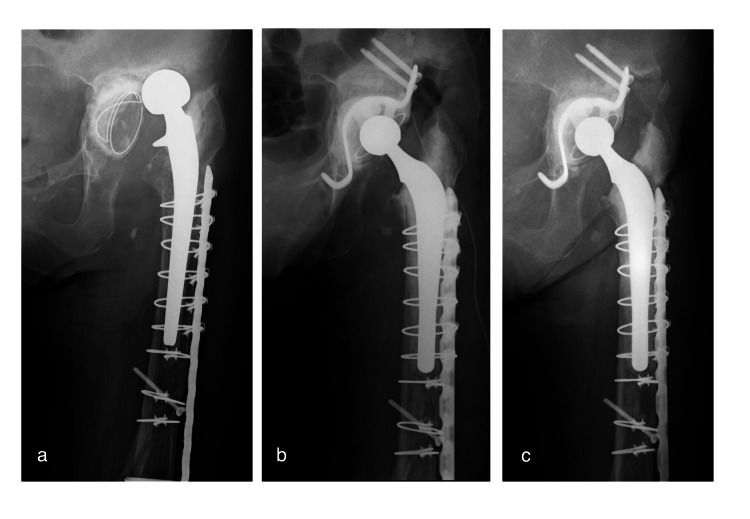
Anteroposterior hip radiographs of an 82-year-old woman who underwent primary THA 14 years ago and revision THA 1 year ago, with rheumatoid arthritis and hemodialysis (case 12, preoperative score of 7 points). (a) Radiograph showing acetabular cup loosening and dislocation due to periprosthetic hip infection; (b) radiograph immediately after one-staged revision THA, including cup and stem; (c) radiograph at four years postoperatively. THA: total hip arthroplasty.

**Figure 5:**
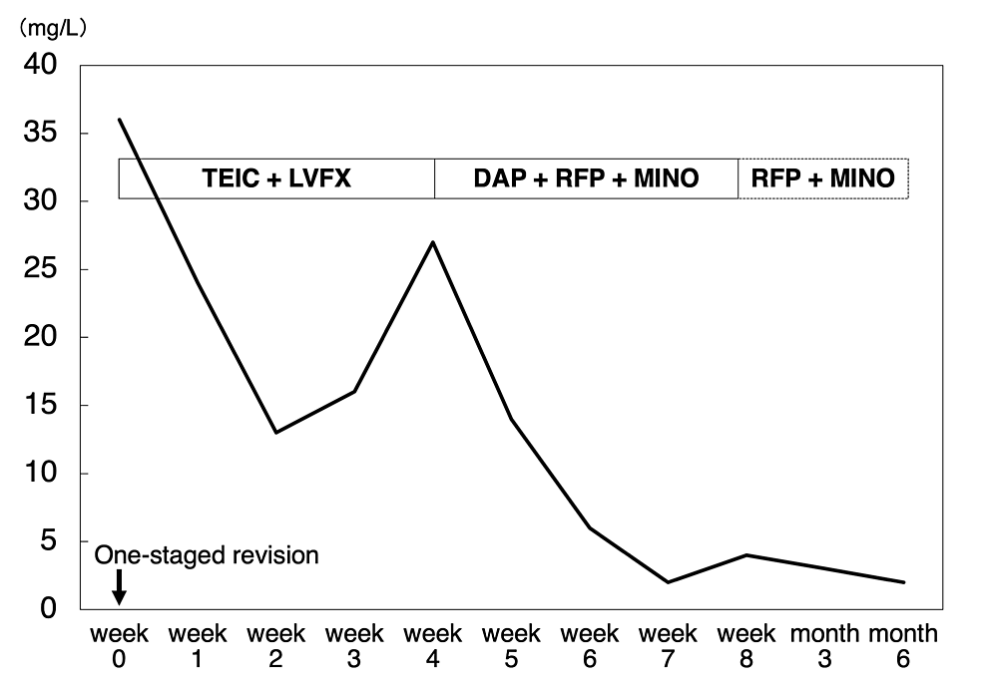
Treatment course and C-reactive protein concentration after DAP therapy in case 12. TEIC: teicoplanin, LVFX: levofloxacin, DAP: daptomycin, RFP: rifampicin, MINO: minocycline hydrochloride.

Additionally, antibiotic conversion to DAP was observed in some cases. A one-staged revision was performed in one patient (case 12) because of acetabular cup loosening and dislocation due to PHI. After DAP therapy, the treatment continued its normal course (Figures 4 and 5).

## Discussion

The frequency of PJIs is increasing, according to the Nordic Arthroplasty Register Association [8], and the treatment of PJIs should target Gram-positive pathogens because such organisms cause the majority of PJIs, and especially those that became antibiotic-resistant [9-12]. In Japan, the proportion of Gram-positive PJIs is over 70%, while 42% of those are methicillin-resistant *S. aureus* (MRSA) [9]. In Europe and the United States, more than 50% of PJIs are typically caused by staphylococcal organisms; and this percentage value is expected to increase [11,12]. The increased frequency of Gram-positive infections and the rise in resistance to commonly used antibiotics have led to the need for novel antibiotic therapies, such as DAP, which has high antimicrobial penetration into biofilms with low bactericidal concentration [13]. According to the European Registry, DAP was effective and safe in patients with osteomyelitis or those with orthopedic device infections; it was also a valuable treatment option for the management of Gram-positive infections [3,14]. However, in the treatment of PJIs by using DAP, there is no consensus on whether the implant should be removed or not.

In the current study, the clinical success rates in the implant removal group were significantly higher than those in the implant retention group. However, the patients without chronic late infection and with a score higher than 7 points did not require implant removal. Tsukayama et al. [5] analyzed the relationship between clinical settings and outcomes in 106 patients with PHIs and reported that all patients with early postoperative infections (success rates: 71%) or acute hematogenous infection (success rates: 50%) had the only debridement, while all patients with late chronic infection (success rates: 85%) were subjected to revision arthroplasty. Therefore, patients with late chronic infections may not be approved for implant retention even if DAP is administered.

Past reports of DAP treatment of PJI are shown in Table 3, and even some authors recommended the use of high-dose of DAP for the treatment of PJI with implant retention [2,15-21]. Furthermore, the European Registry demonstrated high clinical success with DAP therapy, including both implant retention (56%) and removal (44%). Additionally, patients receiving both DAP and RFP showed higher success rates than those who did not concomitantly receive RFP [3]. Interestingly, in vitro experiments showed that DAP had the fastest eradication rate for MRSA embedded in a biofilm; therefore, the combination of DAP and RFP may be a promising treatment option for implant-associated MRSA infections [22,23]. Moreover, the combination of high-dose DAP (equivalent to 8-10 mg/kg/day in humans) and RFP was highly effective for the treatment of foreign body-related MRSA infections [24,25]. Lora-Tamayo et al. [19] also analyzed 18 Staphylococcal PJIs in a multicenter study and concluded that high-dose DAP (10 mg/kg/day) plus RFP was a good initial treatment for PJIs with implant retention. In the current study, however, the mean daily dose of DAP for successful cases of implant retention was not always high, although RFP was administered whenever possible. Either way, RFP may be an important addition to consider when treating PJIs with DAP, with or without implant removal.

**Table 3:** Past reports of daptomycin treatment of periprosthetic joint infections.

Author	Year published	Number of joints	Daily dose of daptomycin (mg/kg/day)	Infection control rates	
				Implant retention	Implant removal
Rao and Regalla [2]	2006	11	4	25% (1/4)	71% (5/7)
Antony et al. [15]	2008	30	6	None	67% (20/30)
Licitra et al. [16]	2010	14	≥6	100% (3/3)	100% (11/11)
Corona Pérez-Cardonaet al. [17]	2012	14	6.6	100% (5/5)	67% (6/9)
Jugun et al. [18]	2013	13	≥8	100% (4/4)	100% (9/9)
Lora-Tamayo et al. [19]	2014	18	10	50% (9/18)	None
Kuo et al. [20]	2016	22	6	None	100% (22/22)
Chang et al. [21]	2017	16	8.3	80% (4/5)	91% (10/11)
Current study		19 (hips)	5.8	67% (6/9)	90% (9/10)

Some limitations of our study must be noted. First, the sample size was small, involving only 20 individuals, due to the difficulties of obtaining a larger patient sample from a single institution. Furthermore, the current study was not performed as a randomized controlled trial. Patients with refractory PHIs required treatment on a case-by-case basis; therefore, the optimal surgical intervention and daily DAP dose also differed. Second, some patients, who did not undergo implant removal after treatment, continued to take other antibiotics orally. As a consequence, because they did not undergo implant removal, they may have actually experienced recurrent infections. Third, we administered DAP when a PHI was suspected prior to the identification of the specific pathogen because such organisms cause the majority of PHIs, and DAP still works in presence of antibiotic resistance. However, such an approach may lead to the administration of excessive levels of antibiotics if treatments need to be switched to a more appropriate antibiotic after pathogen identification.

## Conclusions

Infection control rates in the implant retention and implant removal groups were 67% and 90%, respectively. Late chronic infection was the infection type for all the failure cases in the implant retention group. Furthermore, in the implant retention group, the mean preoperative risk score for the successful cases was significantly higher than that for the failure cases. Patients with low-risk, who did not present chronic late infection but received a preoperative risk score higher than 7 points, may not require implant removal, suggesting that DAP may be a breakthrough alternative to traditional PHI management.
